# Transcriptome Sequencing Data Sets for Determining Gene Expression Changes Mediated by Phase-Variable DNA Methyltransferases in Nontypeable Haemophilus influenzae Strains Isolated from Patients with Chronic Obstructive Pulmonary Disease

**DOI:** 10.1128/MRA.00526-19

**Published:** 2019-07-18

**Authors:** John M. Atack, Timothy F. Murphy, Melinda M. Pettigrew, Kate L. Seib, Michael P. Jennings

**Affiliations:** a Institute for Glycomics, Griffith University, Gold Coast, Queensland, Australia; b Clinical and Translational Research Center, University at Buffalo, State University of New York, Buffalo, New York, USA; c Yale School of Public Health, New Haven, Connecticut, USA; Indiana University, Bloomington

## Abstract

Nontypeable Haemophilus influenzae (NTHi) is a major bacterial cause of exacerbations in chronic obstructive pulmonary disease (COPD). Here, we report high-depth coverage transcriptome sequencing (RNA-seq) data from two NTHi strains, each encoding a different phase-variable methyltransferase. *modA* phase variation results in gene expression differences. These data will serve as an important resource for future studies.

## ANNOUNCEMENT

Nontypeable Haemophilus influenzae (NTHi) is a human-adapted pathogen that causes exacerbations in chronic obstructive pulmonary disease (COPD) (1). Globally, COPD affects ∼65 million people (2). Previous work examining NTHi pathobiology showed that phase-variable *modA* genes, encoding DNA methyltransferases, are involved in virulence by epigenetically regulating multiple genes (3–6). These systems are called phase-variable regulons (phasevarions) (7, 8). ModA allelic variants are defined by variation (<25% identity between alleles) in their central target recognition domain (TRD). The TRD determines methyltransferase specificity (9). Therefore, alleles containing different TRDs methylate different DNA sequences (7) and would control different phasevarions. Examination of an extensive panel of NTHi isolates from COPD patients (10) revealed the presence of two novel *modA* alleles, namely, *modA15* and *modA18*. We previously demonstrated that these methyltransferases are phase-variably expressed in prototype strains (11). Using matched *modA* on-off pairs of each of these prototype strains, we prepared triplicate biological replicates of total RNA using TRIzol (Thermo Fisher) according to the manufacturer’s instructions. NTHi strains were grown to mid-log phase in brain heart infusion (BHI) broth (Oxoid, UK) at 37°C aerobically with 150 rpm shaking.

All methods used for transcriptome sequencing (RNA-seq) are identical to those described previously (12), except we used 100-bp paired-end sequence reads. Briefly, the Illumina Ribo-Zero Gold kit was used to prepare libraries, and libraries were assessed using an Agilent Bioanalyzer DNA 1000 chip. Quantitative PCR (qPCR) quantification assessed each library prior to normalizing (2 nM) and pooling using the Illumina cBot system and TruSeq paired-end (PE) cluster kit v3 reagents. The Illumina NovaSeq system with TruSeq sequencing by synthesis (SBS) kit v3 reagents was used to perform sequencing. Cleaned sequence reads were aligned against the respective reference genomes (GenBank accession numbers CP029620 [10P129H1; *modA15*] and CP029621 [84P36H1; *modA18*]). Reads were mapped to the respective genome using Bowtie2 aligner (v2.3.3.1), and transcripts were assembled with the read alignment and reference annotation-based assembly option in Stringtie v1.3.3 (http://ccb.jhu.edu/software/stringtie/). The Gencode annotation containing both coding and noncoding sequences for each genome was used to map reads (http://www.gencodegenes.org/). Raw gene count values were analyzed with edgeR (https://bioconductor.org/packages/release/bioc/html/edgeR.html), and the subread package (http://subread.sourceforge.net/) was used to quantitate counts using the featureCounts v1.5.3 utility. Gene expression differences were expressed as log2 fold change of expression (logFC). Analysis also generated the following: average log count/million for each gene from all samples (logCPM values), quasi-likelihood F statistic for each gene from all samples (F values), *P* values to determine statistically different expression, and the false-discovery rate/adjusted *P* value for multiple-hypothesis testing.

Multiple genes are differentially regulated commensurate with *modA15* and *modA18* phase variation (44 genes differentially regulated between *modA15* on versus off; 10 genes differentially regulated between *modA18* on versus off). For example, the universal stress protein UspA (DLJ98_08875) and the cold shock protein CspD (DLJ98_02355) are both upregulated when *modA15* is off. In the ModA18 phasevarion, two hypothetical genes (DLK00_05505 and DLK00_05510), encoding proteins of unknown function, are upregulated when *modA18* is on. These RNA-seq data will serve as a primary resource for studying the regulatory events resulting from genome-wide methylation differences by phase-variable methyltransferases during COPD and for determining the exact molecular mechanisms of *modA*-mediated gene expression differences.

### Data availability.

These data have been deposited in the NCBI Gene Expression Omnibus under the accession numbers GSE129761 (*modA15* on versus off) and GSE129764 (*modA18* on versus off).

## References

[1] Murphy TF, Brauer AL, Schiffmacher AT, Sethi S 2004 Persistent colonization by Haemophilus influenzae in chronic obstructive pulmonary disease. Am J Respir Crit Care Med 170:266–272. doi:10.1164/rccm.200403-354OC. 15117742

[2] Sethi S, Murphy TF 2008 Infection in the pathogenesis and course of chronic obstructive pulmonary disease. N Engl J Med 359:2355–2365. doi:10.1056/NEJMra0800353. 19038881

[3] Srikhanta YN, Maguire TL, Stacey KJ, Grimmond SM, Jennings MP 2005 The phasevarion: a genetic system controlling coordinated, random switching of expression of multiple genes. Proc Natl Acad Sci U S A 102:5547–5551. doi:10.1073/pnas.0501169102. 15802471 PMC556257

[4] Atack JM, Srikhanta YN, Fox KL, Jurcisek JA, Brockman KL, Clark TA, Boitano M, Power PM, Jen FEC, McEwan AG, Grimmond SM, Smith AL, Barenkamp SJ, Korlach J, Bakaletz LO, Jennings MP 2015 A biphasic epigenetic switch controls immunoevasion, virulence and niche adaptation in non-typeable *Haemophilus influenzae*. Nat Commun 6:7282. doi:10.1038/ncomms8828. 26215614 PMC4525171

[5] Brockman KL, Branstool MT, Atack JM, Robledo-Avila F, Partida-Sanchez S, Jennings MP, Bakaletz LO 2017 The ModA2 phasevarion of nontypeable *Haemophilus influenzae* regulates resistance to oxidative stress and killing by human neutrophils. Sci Rep 7:3161. doi:10.1038/s41598-017-03552-9. 28600561 PMC5466613

[6] Brockman KL, Jurcisek JA, Atack JM, Srikhanta YN, Jennings MP, Bakaletz LO 2016 ModA2 phasevarion switching in nontypeable *Haemophilus influenzae* increases the severity of experimental otitis media. J Infect Dis 214:817–824. doi:10.1093/infdis/jiw243. 27288538 PMC4978376

[7] Atack JM, Tan A, Bakaletz LO, Jennings MP, Seib KL 2018 Phasevarions of bacterial pathogens: methylomics sheds new light on old enemies. Trends Microbiol 26:715–726. doi:10.1016/j.tim.2018.01.008. 29452952 PMC6054543

[8] Tan A, Atack JM, Jennings MP, Seib KL 2016 The capricious nature of bacterial pathogens: phasevarions and vaccine development. Front Immunol 7:586. doi:10.3389/fimmu.2016.00586. 28018352 PMC5149525

[9] Gawthorne JA, Beatson SA, Srikhanta YN, Fox KL, Jennings MP 2012 Origin of the diversity in DNA recognition domains in phasevarion associated *modA* genes of pathogenic *Neisseria* and *Haemophilus influenzae*. PLoS One 7:e32337. doi:10.1371/journal.pone.0032337. 22457715 PMC3311624

[10] Pettigrew MM, Ahearn CP, Gent JF, Kong Y, Gallo MC, Munro JB, D’Mello A, Sethi S, Tettelin H, Murphy TF 2018 *Haemophilus influenzae* genome evolution during persistence in the human airways in chronic obstructive pulmonary disease. Proc Natl Acad Sci U S A 115:E3256–E3265. doi:10.1073/pnas.1719654115. 29555745 PMC5889651

[11] Atack JM, Murphy TF, Bakaletz LO, Seib KL, Jennings MP 2018 Closed complete genome sequences of two nontypeable *Haemophilus influenzae* strains containing novel *modA* alleles from the sputum of patients with chronic obstructive pulmonary disease. Microbiol Resour Announc 7:e00821-18. doi:10.1128/MRA.00821-18. 30533802 PMC6211359

[12] Atack JM, Bakaletz LO, Jennings MP 2019 High-depth RNA-seq data sets for studying gene expression changes mediated by phase-variable DNA methyltransferases in nontypeable Haemophilus influenzae. Microbiol Resour Announc 8:e01500-18. doi:10.1128/MRA.01500-18. 30643897 PMC6328670

